# Quantitative study of the capillaries within the white matter of the Tg2576 mouse model of Alzheimer's disease

**DOI:** 10.1002/brb3.1268

**Published:** 2019-03-21

**Authors:** Yi Zhang, Feng‐lei Chao, Lei Zhang, Lin Jiang, Chun‐ni Zhou, Lin‐mu Chen, Wei Lu, Rong Jiang, Yong Tang

**Affiliations:** ^1^ Department of Laboratory Medicine, Key Laboratory of Diagnostic Medicine, Ministry of Education Chongqing Medical University Chongqing China; ^2^ Department of Histology and Embryology Chongqing Medical University Chongqing China; ^3^ Laboratory of Stem Cells and Tissue Engineering Chongqing Medical University Chongqing China

**Keywords:** Alzheimer's disease, capillary, Morris water maze, stereology, Tg2576, white matter

## Abstract

**Introduction:**

To quantitatively investigate the capillaries within the white matter of Tg2576 Alzheimer's disease (AD) transgenic mice during the early stage.

**Methods:**

In the current study, 10‐month‐old male Tg2576 AD mice were used as the early‐stage AD group and age‐matched nontransgenic littermate mice were used as the wild‐type group. Then, the Morris water maze was used to examine the spatial learning and memory abilities of the mice in both groups, and unbiased stereological methods were used to accurately quantify the volume of white matter and the parameters of the capillaries within the white matter, such as the total length, total volume, and total surface area of capillaries.

**Results:**

The Morris water maze performance of the Tg2576 group was worse than that of the wild‐type group, while the white matter volume did not significantly differ between the wild‐type group and the Tg2576 group. The total length, total volume, and total surface area of the capillaries within the white matter of the Tg2576 group were significantly decreased compared to those of the wild‐type group.

**Conclusions:**

The current study provide structural basis for understanding the pathological changes of the early stage of AD and cognitive decline in AD might be associated with changes in the white matter capillaries. Capillaries within the white matter might, thus, serve as a valid target for the prevention and treatment of early‐stage AD.

## 1 INTRODUCTION

Alzheimer's disease (AD) is a common neurodegenerative disease in clinical settings that is characterized by progressive memory deficits and cognitive disorder (Querfurth & LaFerla, 2010). Currently, more than 40 million people worldwide suffer from AD. One in nine people who are aged more than 65 years of age suffered from AD, and one‐third of people 85 years of age and older have AD (Alzheimer's Association, 2015). Due to the serious physical and psychological impacts of memory dysfunction on AD patients and the low living quality of families, AD has become a widely recognized social and health concern. Studies of the pathogenesis occurring during early AD are very critical and urgently needed (Goedert & Spillantini, 2006). Over the past 20 years, several hypotheses have been proposed regarding the pathogenesis of AD. Among them, the amyloid hypothesis is the most accepted and investigated. However, the exact role of Aβ in the pathogenesis and pathological process of AD is still not entirely clear, and it remains controversial with regard to whether the amyloid hypothesis itself is valid or not. Moreover, the amyloid hypothesis has been challenged by the vascular hypothesis (Clark et al., 2017; Farkas & Luiten, 2001; Østergaard et al., 2013; Zlokovic, 2005). Currently, the findings of an increasing amount of researches show that vascular pathological changes might play a key role in the pathogenesis and pathological process of AD (van Norden et al., 2012; de la Torre, 2002a). Capillaries, the thinnest and most widely distributed blood vessel, play a vital role role in maintaining cerebral blood flow (CBF) and regulating brain microcirculation. Studies have shown that pathological changes in capillaries in the brain play an extremely important role in the pathogenesis of this devastating disease (Østergaard et al., 2013; de la Torre, 2002b). Previous studies have reported a wide variety of vascular pathological changes in the AD brain. Cerebral capillary degeneration, included looping, twining, and braiding of vessels, has been shown to be present in pathologically confirmed cases of AD (Farkas & Luiten, 2001; Østergaard et al., 2013). These pathological changes have been shown to cause brain capillary microcirculation deficits and brain hypoperfusion, which could lead to both cognitive and memory decline in AD (Attems & Jellinger, 2004; Oshima et al., 2006).

The pathological changes in brain microvessels play a crucial role in the pathogenesis of AD. There are still questions regarding the brain regions where these microvessel changes might be most relevant to the pathogenesis of AD. People take it for granted that the pathological changes in the brain microvessels of the hippocampus decisive in AD because the hippocampus mainly responsible for storage conversion of long‐term memory. However, Buѐe et al's study showed that the microvascular density of the cerebral cortex was significantly decreased in Alzheimer's disease patients compared to age‐matched controls (Buѐe et al., 1994). Moreover, Moody et al. reported that vascular changes were more often associated with white matter lesions than other regions of the brain because the capillary distribution within the white matter is relatively smaller; thus, the white matter is more susceptible to hypoxic‐ischemic injury (Moody, Brown, Challa, Ghazi‐birry, & Reboussin, 1997). Recently, the findings show that, in the early stages of AD, both cognitive and memory decline are closely associated with damage to the structural integrity of white matter (Bendlin et al., 2010; Huang, Friedland, & Auchus, 2007; Rémy, Vayssière, Saint‐Aubert, Barbeau, & Pariente, 2015; Zhang et al., 2013). Using clinical diffusion tensor imaging (DTI) or magnetic resonance imaging (MRI), neuroimaging studies found that ischemic changes in white matter existed in mild cognitive impairment (MCI) and AD patients (Jones et al., 1999; Medina et al., 2006). Moreover, the white matter is more susceptible to hypoxic‐ischemic injury (Van Gijn, 1998; Moody et al., 2004; Nonaka et al., 2003). Van Gijin thought that vascular factors might be closely related to the damage in white matter (Van Gijn, 1998). Capillaries can be easily identified by the diameter size and complex network structure by using brain transparency technology, immunohistochemistry staining, and scanning electron microscopy to observe vascular corrosion models. In a study of 109 human brains, Nonaka et al. observed the phenomena in which the blood vessels in the white matter coiled, looped, and spiraled, while the blood vessels ran straight through the cortex (Nonaka et al., 2003). Previous studies have reported that spiral‐like blood vessels and periventricular venous collagenosis, which result in vascular contortion and occlusion, are usually related to white matter lesions in AD (Brown, Moody, Thore, & Challa, 2000). Thus, capillary microcirculation changes may be a critical structural basis for white matter lesions in AD. Therefore, the capillary pathological changes have been thought to be a crucial source of cognitive impairment in AD (Farkas & Luiten, 2001; van Norden et al., 2012; Salat et al., 2009; de la Torre, 2002a, 2002b). However, no study has quantified the capillary pathological changes in the white matter of early stage in AD patients or AD models.

In this study, we used the Morris water maze behavior test to investigate spatial learning and memory abilities, and then used accurate three‐dimensional quantitative stereological methods to investigate the white matter and the parameters of the capillaries within the white matter of 10‐month‐old Tg2576 AD mice. The present study could not only provide an accurate quantitative methodological design for future studies on the capillaries changes in the AD white matter, but also provide a structural basis for capillary pathological changes in the white matter during early‐stage AD.

## 2 METHODS

### 2.1 Animals

Eleven Tg2576 AD male mice (Overexpress the Swedish mutation of human amyloid precursor protein, APP695swe) and 11 nontransgenic littermate male mice (wild‐type) were selected randomly at 10 months of age.

All mice were raised by experimental animal center of Chongqing Medical University, P. R. China. All mice were reared in a standard environment, and the animal experiment followed the National Institute of Health's guide for the care and use of laboratory animals.

### 2.2 Genetic identification

Tg2576 AD mice is short for Tg(HuAPP695SWE)C57B6 × C57B6/SJL‐2576. The primer sequences are shown below.

Tg(APPswe) sequence：transgene = ~ 350 bp

oIMR: 5′ ctg acc act cga cca ggt tct ggg t 3′

oIMR: 5′ gtg gat aac ccc tcc ccc agc cta gac ca 3′

DNA extraction: The mouse tail tip was cut and incubated with proteinase K overnight and the DNA was extracted with a genomic extraction reagent.

PCR protocol: First, a 25 μl PCR system was prepared with 9 μl ultrapure water +0.5 μl upstream primer +0.5 μl downstream prime +2.5 μl DNA template (extracted) +12.5 μl 2 × premixed Taq PCR mix. Then, the target DNA samples were added and PCR amplification was performed by using the PCR cycle program. The PCR cycle program included predenaturation at 94°C for 3 min, denaturation at 94°C for 45 s, annealing at 56°C for 60 s, extension at 72°C for 60 s, for a total of 30 cycles. Finally, the DNA samples were stored at 4°C.

Electrophoresis: 1.5% agarose gel was prepared, and 3 μl target DNA and DNA maker in each panel were added. Electrophoresis at 90V for 30 min. After the end of electrophoresis, the gel was observed in the gel imaging system.

### 2.3 Morris water maze

The Morris water maze test was used to measure the spatial learning and memory abilities of the mice (Chen et al., 2000; Morris, 1984). In this study, the test lasted seven consecutive days and included two parts. Part one was the hidden platform test and was conducted within 1–6 days. A platform with a fixed position was placed ~1 to 1.5 cm underwater, with every test time up to 60 s, and the escape latency (the real‐time mouse find the platform) was recorded as an evaluation of spatial learning and memory capabilities. Part two was the probe trial test, which was conducted on the seventh day. The probe trial provided an index of the mice tendency to persist around the platform's previous location and is generally considered to be a measure of retention memory. After completion of the hidden platform test, the original platform was removed, each mouse was given 60 s to freely swim, and the frequency of target zone crossings and the percentage of time spent in the target quadrant zone were recorded to evaluate spatial exploration (Chao et al., 2018).

### 2.4 Brain preparation

**Figure 1:**
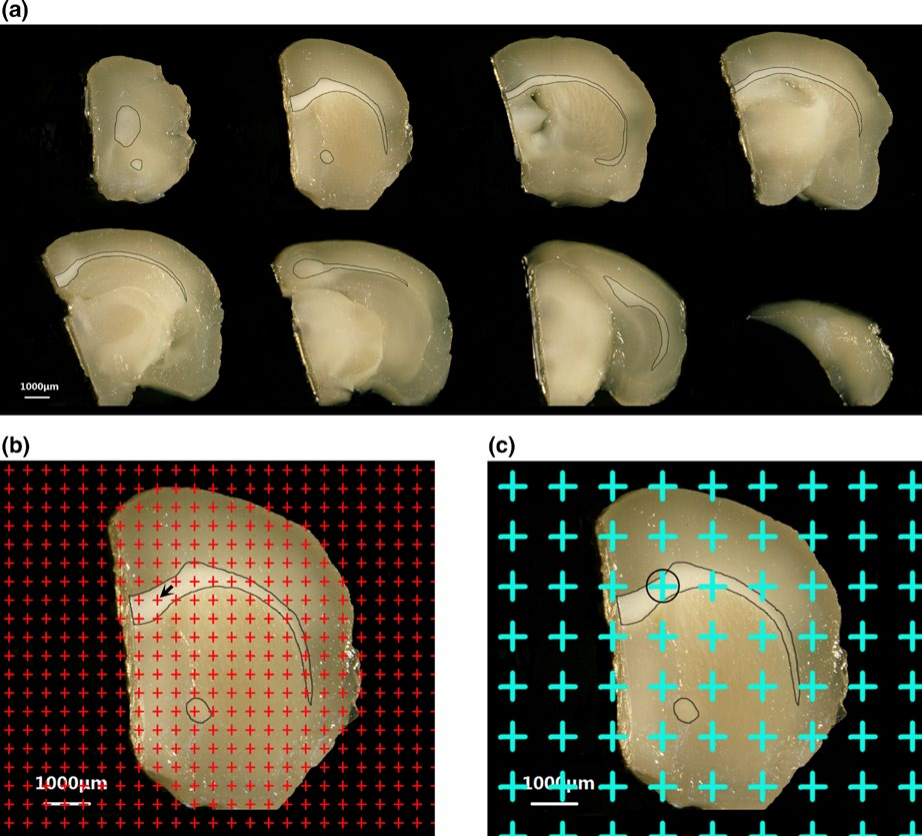
The stereological methods. (a) The successive 1 mm cerebral slices of one hemisphere and the boundary of the white matter of each slice. Bar = 1,000 μm. (b) The point grid is randomly placed on successive equidistant brain slices, and the number of the points hitting the white matter are counted. The arrow (←) indicates one of the counted points. Bar = 1,000 μm. (c) The point grid is randomly superimposed on the slice, and those points hitting the white matter are sampled. ○ shows the point hitting the white matter. Bar = 1,000 μm

The mice were weighed and anaesthetized with 0.4 ml/100 g pentobarbital sodium and then perfused with 0.9% sodium chloride plus heparin followed by 4% paraformaldehyde. After the perfusion, the cerebral hemispheres were removed. Then, the hemispheres were embedded with 6% agar in the Mouse Brain Matrices (Rayward life technology co. LTD; Shenzhen, China). Using knives to cut each hemisphere into continuous 1‐mm‐thick coronal slices (Figure 1a).

### 2.5 Estimation of the white matter volume

The caudal surface of each 1‐mm‐thick coronal slice was photographed using anatomical microscope. Then, an equidistant counting grid probe was superimposed at random onto each captured photograph, and the points hitting the white matter were counted (Figure 1b). The total volume of the white matter was calculated according to Cavalieri's estimator (Tang, Nyengaard, Pakkenberg, & Gundersen, 1997; Zhang et al., 2017).

(1) Vwm=t×a(p)×∑P(wm)

where *V*wm means the total volume of the white matter, a (*p*) means the area represented by each measuring point of the equidistant counting grid probe (0.59 mm^2^), *t* means the thickness of coronal slice (1 mm), and *ΣP* (wm) means the total number of counting points hitting the white matter.

### 2.6 White matte tissue sampling and frozen sections

A transparent equidistant counting grid was superimposed at random onto each 1‐mm‐thick coronal slice. Based on the points that hit the white matter, three or four tissue blocks were randomly sampled (Figure 1c). After being postfixed, dehydrated, and embedded, the tissue blocks were sectioned at 4 μm in four different planes, randomly. Using the isector technique, the sampled tissue was cut into isotropic sections, which assumed that the capillaries had the same probability of being sampled in every direction of 3D space (Nyenggard & Gundersen, 1992).

### 2.7 Immunohistochemical procedures

Using the streptavidin–peroxidase (SP) method, the immunohistochemistry was performed by several steps. Briefly, the sections were fixed with acetone at 4°C for 10 min, and pretreated with 0.01 M citrate buffer solution at 99°C for 20 min for antigen retrieval, and then the sections were pretreated with 3% hydrogen peroxide at 25°C for 15 min and incubated with the working solution of normal goat serum for 30 min at 37°C. After washing thrice with PBS, a 1:500 dilution of anti‐mouse CD31 primary antibody (Abcam, Cambridge, UK) was prepared and used on labeled vascular endothelial cells (Balcells et al., 2010; Cheung et al., 2015; Nojiri et al., 2015; Vecchi et al., 1994). The slices were overnight incubated with CD31 work solutions at 4°C. The next day, after rewarming and washing, the goat anti‐mouse immunoglobulin G secondary antibody working solution was added to the slices at 37°C for 30 min. Followed by washing, the sections were incubated at 37°C for 30 min with a streptavidin–horseradish peroxidase working solution. After this procedure, staining was developed with diaminobenzidine (DAB) for 60 s. In the end, the sections were dehydrated with graded ethanol, transparentized in graded dimethylbenzene alcohol solution, and mounted with neutral resins.

### 2.8 Immunofluorescence procedures

The sections of both group mice were rinsed for 4 × 15 min in PBST. After repairing in boric acid solution for 20 min, nonspecific binding sites were blocked by incubating the sections with normal goat serum for 2 hr at 37°C. The primary antibodies anti‐beta amyloid (mouse, ab11132, Abcam) were then added at a dilution of 1:500 in PBS and incubated at 4°C for 72 hr and then rewarmed at 37°C for 1 hr. The secondary antibodies (1:200) DyLight 488 (Abbkine) were then added for 2 hr, followed with DAPI chemical staining. Finally, the sections were mounted on slides. The slides were then viewed and photographed under fluorescence microscope.

### 2.9 Stereological sampling

The immunohistochemical sections were viewed and photographed using a modified microscope. The boundaries of the slice were drawn by using a low magnification objective lens (4×), Then, using an oil objective lens (100×), the images were captured by stereological analysis software (Glostrup, Denmark). Blood vessels with diameter less than 10 μm were defined as capillaries and were used for the stereological analysis (Alba, Vidal, Díaz, Villena, & Vargas, 2004).

### 2.10 Stereology analyses

#### 2.10.1 Estimation of the total length of the capillaries in the white matter

**Figure 2:**
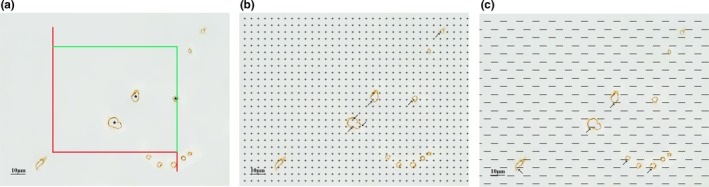
(a) An unbiased counting frame is randomly placed on the view of white matter, and the capillary profiles inside the counting frame and only crossing the counting lines (green lines) rather than the prohibiting lines (red lines) are counted. ★ shows counted capillary profiles. Bar = 10 μm. (b) The point grid is randomly placed on the view of white matter, and the grid points inside the capillaries(the standard is the top right of the cross point inside the capillaries) was recorded. Bar = 10 μm. The arrows (→) indicate the counted points. (c) Test lines are put on the view of white matter, and the number of intersections between the test lines and capillary luminal surfaces are counted. Bar = 10 μm. The arrows ( →) indicate the counted intersections between the test lines and the capillaries

An unbiased counting frame was randomly superimposed onto each captured photograph (Tang & Nyengaard, 1997). The capillary profiles inside the counting frame or touching inclusion lines (the top line or right line) were counted (Figure 2a). The capillary length density in the white matter was calculated according to the following formula (Qiu et al., 2011; Shao et al., 2010; Tang & Nyengaard, 1997; Tang et al., 1997; Zhang et al., 2017).

(2) $$Lv\left(cap/\text{wm}\right)=2\times \frac{\sum Q(cap)}{\sum A}$$

where *Lv* (*cap/*wm) is the capillary length density in the white matter, Σ*Q* (*cap*) is the total number of the capillary profiles counted per white matter sections, and Σ*A* is the total area of the unbiased counting frames used in the white matter. The capillary total length in the white matter then equaled the value of capillary length density in the white matter multiplied by the total volume of white matter per mouse.

#### 2.10.2 Estimation of the total volume of the capillaries in the white matter

A equidistant counting grid probe was randomly superimposed onto each captured photograph. The numbers of test points hitting the white matter capillaries and the white matter were respectively counted (Figure 2b). The capillary volume density in the white matter was calculated according to the following formula (Zhang et al., 2017).

(3) $$Vv\left(cap/\text{wm}\right)=\frac{\sum P(cap)}{\sum P(\text{wm})}$$

where *Vv*(*cap/*wm) is the capillary volume density in the white matter, *ΣP* (*cap*) is the total number of counting points hitting the capillaries in the white matter, and *ΣP* (wm) is the total number of counting points hitting the white matter.

The capillary total volume in the white matter equaled the value of capillary volume density in the white matter multiplied by the total volume of white matter per mouse.

#### 2.10.3 Estimation of the total surface area of the capillaries in the white matter

A test line probe was randomly superimposed onto each captured photograph. The intersection numbers of the test lines and the luminal surface of the capillaries were counted, and the total test line length in the white matter was recorded (Figure 2c). The capillary surface area density in the white matter was calculated according to the following formula (Zhang et al., 2017).

(4) $$Sv\left(cap/\text{wm}\right)=2\times \frac{\sum I(cap)}{\sum L}$$

where *Sv*(*cap/*wm) is the capillary surface area density in the white mattter, *ΣI* (*cap*) is the total number of intersections between capillary lumens and test lines, and *ΣL* is the total test lines length in the white matter.

The capillary total surface area in the white matter equaled the value of capillary surface area density in the white matter multiplied by the total volume of white matter per mouse.

### 2.11 Estimation of the correlations between behavioral results and stereological measurements of the capillaries results

We analyzed the Pearson correlation coefficient to investigate the relationship between spatial learning abilities and stereological data respectively. The escape latency values of the behavioral results (swimming time) of the mice sampled for stereological analyses was adjusted by the stereological measurements of the capillaries (the total length, total volume, and total surface area of the capillaries). And the Pearson correlation coefficient was calculated to investigate the relationship between spatial learning and the stereological results.

### 2.12 Statistics

The behavioral results are presented as the mean ± standard error (*SEM*). The stereological quantitative results are presented as the mean ± standard deviation (*SD*). Statistical analyses were performed using SPSS (ver. 19.0, SPSS Inc, Chicago, IL). The Shapiro–Wilk test was used to evaluate whether the group means of the data from the behavioral test and the stereological quantitative analysis were normally distributed. The escape latency data from the hidden platform task were analyzed using repeated‐measures analysis of variance (ANOVA). The data from the probe trial task and the stereological data were statistically tested by unpaired Student's *t* test. The relationship between behavioral test and the stereological results was calculated by Pearson correlation coefficient. A *p* value <0.05 was adopted throughout the analysis (Chao et al., 2018).

## 3 RESULTS

### 3.1 Morris water maze behavior results

#### 3.1.1 Spatial learning ability

**Figure 3:**
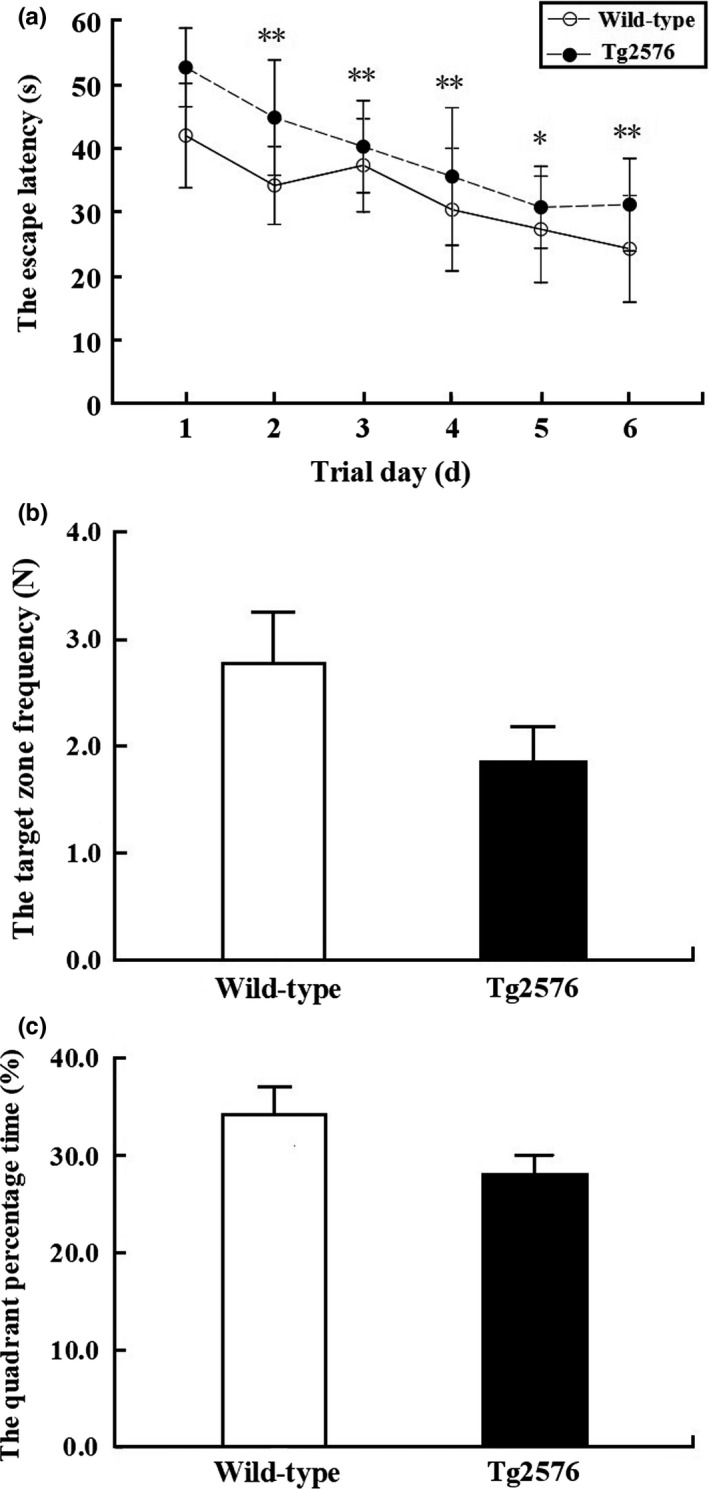
The Morris water maze behavior results. (a) The escape latency of Morris water maze positioning navigation test in 10‐month‐old wild‐type AD mice, Tg2576 AD mice is shown. Each point represents the average of the four escape latencies (x ± sem). * indicates *p* < 0.05. ** indicates *p* < 0.01. (b) The target zone frequency in target quadrant zone of in wild‐type AD mice and Tg2576 AD mice(x ± sem.). (c) The percentage of time in target quadrant zone of wild‐type AD mice and Tg2576 AD mice (x ± sem)

In the hidden platform test, the escape latency of the Tg2576 group mice was significantly longer than that of the wild‐type group mice (*p* < 0.05) (Figure 3a).

#### 3.1.2 Spatial memory abilities

In the probe trial test, there was no significant difference in the cross platform frequency between the Tg2576 group mice and the wild‐type group mice (*p* > 0.05) (Figure 3b). There was no significant difference between the Tg2576 group mice and the wild‐type group mice in the quadrant percentage time (*p* > 0.05) (Figure 3c).

### 3.2 Total volume of the white matter

**Figure 4:**
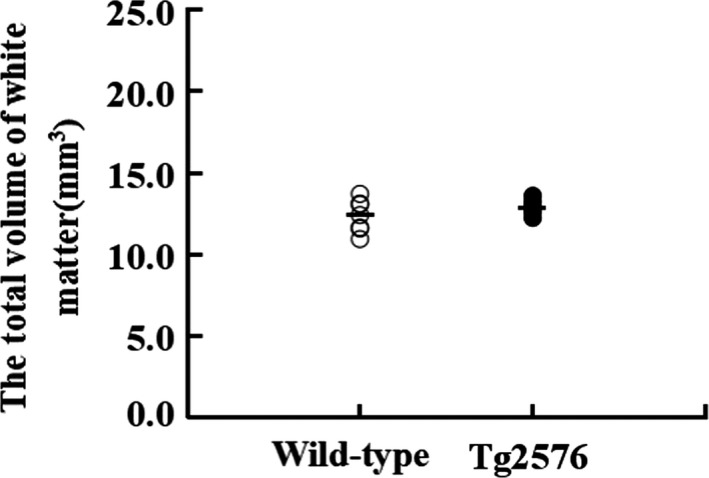
The total volume of the white matter of wild‐type AD mice and Tg2576 AD mice (x ± *SD*)

The total white matter volume of the wild‐type group mice was 12.87 ± 0.44 mm^3^, and the total white matter volume of the Tg2576 group mice was 12.33 ± 1.01 mm^3^. The statistical results indicated that the white matter volume between the Tg2576 group mice and the wild‐type group mice was not significant difference (*p* > 0.05) (Figure 4).

### 3.3 Immunofluorescent results of Aβ

**Figure 5:**
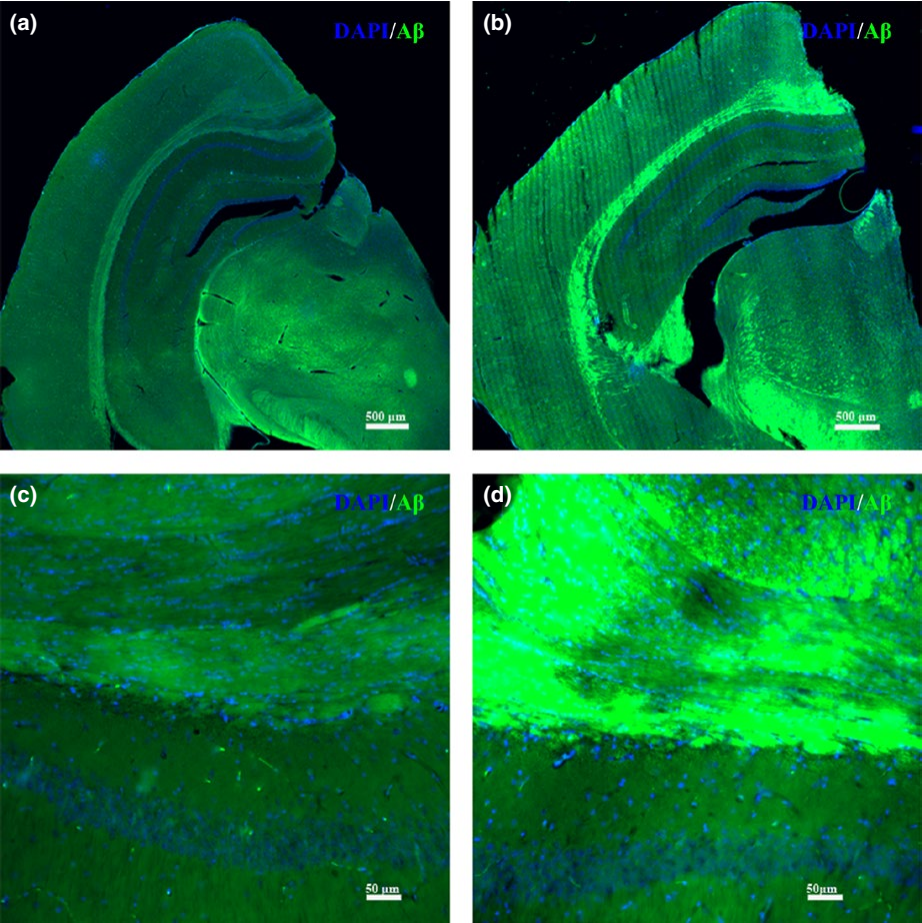
The morphology of Aβ immunofluorescence in the white matter of both groups of mice. (a) The immunofluorescence morphology of the wide‐type mice at 25 times. (b) The immunofluorescence morphology of the Tg2576 transgenic AD mice at 25 times. Bar = 500 μm. (c) The immunofluorescence morphology of the wide‐type mice at 200 times. (d) The immunofluorescence morphology of the g2576 transgenic AD mice at 200 times. Bar = 50 μm

The results of the immunofluorescent‐stained Aβ in the white matter of both groups of mice are shown in Figure 5. High‐intensity Aβ fluorescence was observed in the white matter of the Tg2576 group mice.

### 3.4 Immunohistochemical results of white matter capillaries

**Figure 6:**
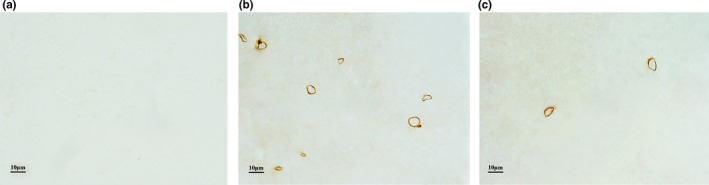
Immunohistochemical results of white matter capillaries. (a) The blank control without primary antibody. (b) The capillaries in the white matter of 10‐month‐old nontransgenic littermates wild‐type mice. (c) The capillaries in the white matter of 10‐month‐old Tg2576 transgenic AD mice. Bar = 10 μm

The results of the immunohistochemical‐stained capillary profiles in the white matter of both groups of mice are shown in Figure 6.

#### 3.4.1 Total length of the capillaries in the white matter

**Figure 7:**
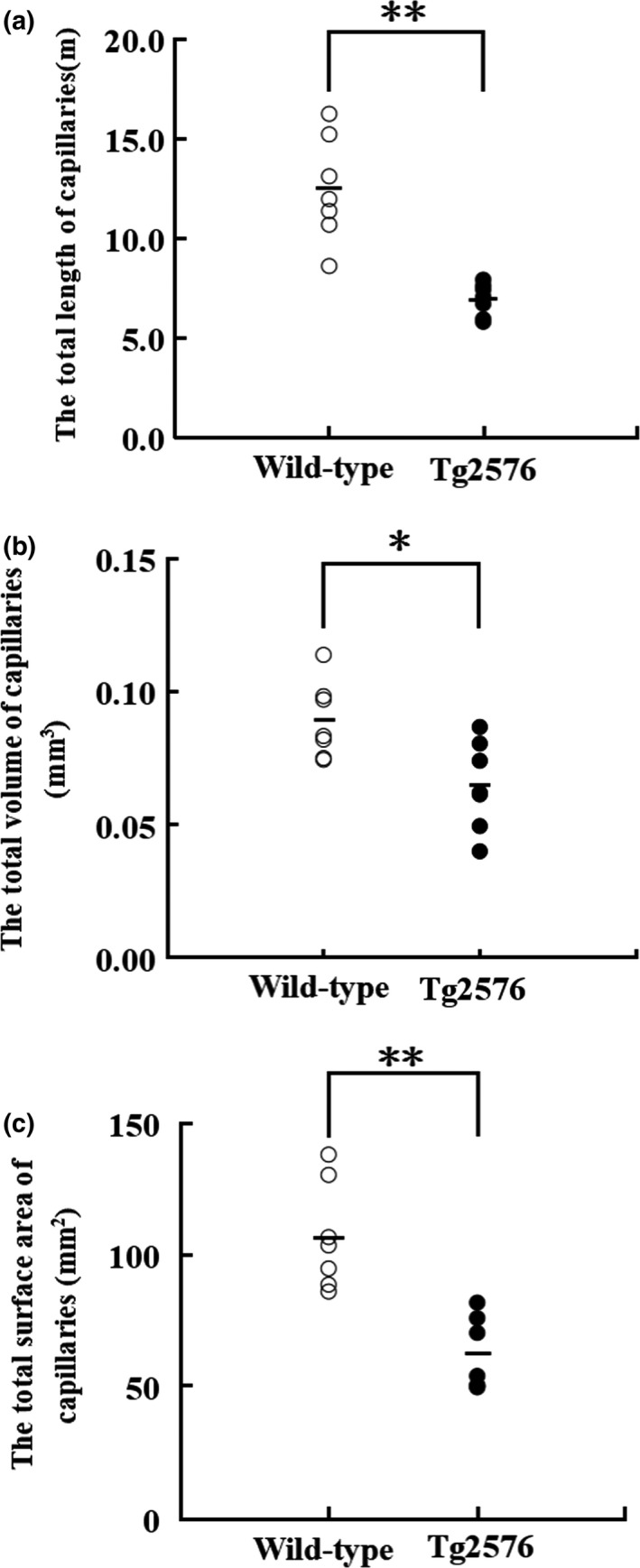
The stereological results of white matter capillaries. (a) The total length of the capillaries in the white matter of wild‐type AD mice and Tg2576 AD mice (x ± *SD*). ** indicates *p* < 0.01. (b) The total volume of the capillaries in the white matter of wild‐type AD mice and Tg2576 AD mice (x ± *SD*). * indicates *p* < 0.05. (c) The total surface area of the capillaries in the white matter of wild‐type AD mice, and Tg2576 AD mice (x ± *SD*). ** indicates *p* < 0.01

The total length of the capillaries in the white matter was 12.44 ± 2.64 m in the wild‐type group mice and 6.90 ± 0.82 m in the Tg2576 group mice. The total length of the capillaries in the white matter of the Tg2576 group mice was significantly shorter than that of the age‐matched wild‐type group mice (*p* < 0.01) (Figure 7a).

#### 3.4.2 Total volume of the capillaries in the white matter

The total volume of the capillaries in the white matter was 0.0891 ± 0.0145 mm^3^ in the wild‐type group mice and 0.0648 ± 0.0167 mm^3^ in the Tg2576 group mice. The total volume of the capillaries in the white matter of the Tg2576 group mice was significantly smaller than that of the age‐matched wild‐type group mice (*p < *0.05) (Figure 7b).

#### 3.4.3 Total surface area of the capillaries in the white matter

The total surface area of the capillaries in the white matter was 106.81 ± 20.17 mm^2^ in the wild‐type group mice and 61.77 ± 13.80 mm^2^ in the Tg2576 group mice. The total surface area of the capillaries in the white matter of the Tg2576 group mice was significantly smaller than that of the age‐matched wild‐type group mice (*p < *0.01) (Figure 7c).

### 3.5 Correlations between behavioral results and capillary results

As shown in Table 1, our data indicated that the all the stereological measurements of the capillaries within the white matter, the total length, total volume, and total surface area of the capillaries, were significantly correlated with the last day escape latency data of the water maze behavioral results both in the wild‐type group and the Tg2576 group (*p* < 0.01) (Table 1).

**Table 1:** Correlations between behavioral tests and stereological measurements

	Escape latency/L (cap, wm)*		Escape latency/V (cap, wm)*		Escape latency/S (cap, wm)*	
	r	p	r	p	r	p
Wild‐type	−0.935	0.002	−0.978	0.000	−0.957	0.001
Tg2576	−0.944	0.001	−0.817	0.025	−0.916	0.004

*Escape latency is the time of Morris water maze positioning navigation test. L (cap, wm) is the total length of the capillaries in the white matter, V (cap, wm) is the total volume of capillaries in the white matter, S(cap, wm) is the total surface area of capillaries in the white matter and r is the correlation coefficient.

## 4 DISCUSSION

Alzheimer's disease (AD) is a central nervous system neurodegenerative disease; however, the pathological changes during the early stage of AD remain inconclusive (Querfurth & LaFerla, 2010). Past studies indicated that brain capillaries play a key role in the onset and progression of cognitive decline in Alzheimer's disease (de la Torre, 2002b,^2004^). However, the quantitative changes in the capillaries in AD brain are unknown. Moreover, it is nearly impossible to investigate the quantitative changes in the capillaries in the human early‐stage AD brain. Therefore, we used a mouse model of early‐stage AD to quantitatively investigate the capillary changes in the early‐stage AD brain.

To study early‐stage AD in mice, the first key is the choice of the right type and the right age of AD mice. Furthermore, appropriate and accurate behavioral tests should be used to detect the typical learning and memory ability decline which are related to the AD. The Tg2576 transgenic mice (APP695swe) are Swedish mutants which could overexpress human amyloid precursor protein. This type of AD mouse is considered one of the ideal models for studying the cellular and molecular pathology of AD in early‐stage Alzheimer's disease (Bilkei‐Gorzo, 2014). Moreover, both the memory and cognitive functions of Tg2576 transgenic AD mice have been shown to decline in association with aging procession (Corcoran, Lu, & Turner, 2002). The Morris water maze is used to study brain function of Alzheimer's disease. It has been widely recognized in the world and is the preferred classical experiment for behavioral research, especially learning and memory research about age‐related memory impairment in transgenic mouse models of AD (Morris, 1984). In this study, the spatial learning and memory abilities of Tg2576 AD mice were detected using the Morris water maze. Previous studies have showed that most of the animal models of AD need at least 6–10 months to begin with deposition of senile plaques or to show learning and memory deficits behavior (Hsiao et al., 1996; Jacobsen et al., 2006; Savonenko et al., 2005; Toledo & Inestrosa., 2010). Savonenko et al. found that, at 6 months of age, APP/PS1 double‐transgenic mice showed visible plaque deposition, but all genotypes, including Tg2576 and APP/PS1, were indistinguishable from the nontransgenic animals in all cognitive measures (Savonenko et al., 2005). Hsiao et al. found that transgenic mice overexpressing APP showed impairments in learning and memory abilities by 9 or 10 months of age (Hsiao et al., 1996). In this study, we used Tg2576 transgenic mice to simulate early‐stage AD, and the Morris water maze was used to test behavior defects in our AD mice. The escape latency of the hidden platform test showed that the spatial learning ability of the 10‐month‐old Tg2576 group mice declined, and the cross platform frequency and quadrant percentage time of the probe trial test showed that the memory ability of the 10‐month‐old Tg2576 group mice declined. All the behavioral tests showed that the behavioral changes occurred in 10‐month‐old Tg2576 AD mice, which was consistent with the results of our previous study, which found that the Tg2576 group mice did not show neurobehavioral changes until 10 months of age (Zhang et al., 2013). The immunofluorescence results of Aβ and our previous ELISA results of soluble Aβ40 and Aβ42 protein from Zhang et al. both indicated the presence of characteristic Aβ deposition of AD in Tg2576 mice (Zhang et al., 2013). Our present findings, together with the findings from previous studies, further suggested that 10‐month‐old Tg2576 AD mice could be meaningfully used to investigate the morphological changes during early‐stage AD.

What might cause the spatial learning and memory ability decline during early‐stage AD? Traditionally, white matter lesions were considered as a secondary pathological event following gray matter damage (Pierpaoli et al., 2001). However, the role of the white matter play in learning and memory ability get more and more attention by researchers. Evidence showed that the volume of white matter were increased after adults learning to read, indicated that altered white matter structure was associated with the learning and cognitive function (Fields, 2010). Alzheimer's disease‐related researches showed that both cognitive and memory decline were associated with white matter lesions (Bendlin et al., 2010; Medina et al., 2006; Salat et al., 2009). In particular, rather than hippocampal atrophy, white matter changes predict incident AD (Brickman et al., 2012). The clinical evidences indicated that there was a lesion in the white matter of the mild cognitive impairment (MCI) and early AD (Rémy et al., 2015; Zhuang et al., 2013). As early as 2000, Brown et al. reported that the spiral‐like blood vessels and periventricular venous collagenosis existed, which caused vascular contortion and occlusion in the white matter in AD (Brown et al., 2000). Later, Jellinger reported that arteriolosclerosis, fibrohyalinosis, lipohyalinosis, and adventitial calcifications were present in the white matter of the AD brain (Jellinger, 2002; Jellinger & Attems, 2003). What might be the relationship between the white matter changes in AD and the capillary changes in the white matter in AD? The vascular supply in the brain was not uniform and the capillary distribution within the white matter is relatively small, and the white matter is more vulnerable than other brain regions to chronic hypoperfusion and hypoxic‐ischemic injury (Van Gijn, 1998; Nonaka et al., 2003), we speculated that capillary microcirculation changes might play a vital role in the white matter lesions in the early‐stage AD. In this study, we used stereological methods to quantitate capillaries within the white matter of 10‐month‐old Tg2576 AD mice. The results showed that the geometric parameters of the capillaries within the white matter of 10‐month‐old Tg2576 AD mice, such as total length, total volume, and total surface area of capillaries in the white matter, were significantly lower than those in the wild‐type mice of the same age. These results indicated that the marked decrease in the capillaries existed in the white matter of Tg2576 AD mice at 10 months of age.

There have been some studies that have investigated the vascular changes in the AD brain (Bourasset et al., 2009; Buѐe, Hof, & Delacourte, 1997; Desai, Schneider, Li, Carvey, & Hendey, 2009; Lee et al., 2005; Paris et al., 2004). In 2004, Paris et al. examine the vascular density and found that the vascular density in the hippocampus and cortex of the APP mutation mice was decreased by approximately 30% compared to control littermates (Paris et al., 2004). Using the vascular space markers, [^3^H]‐inulin and [^14^C]‐sucrose, for in situ brain perfusion, Bourasset reported that the cerebrovascular volume was significantly lower (−26% and −27%) in the brains of 3xTg‐AD mice than in those of nontransgenic littermates (Bourasset et al., 2009). In contrast to the above studies, Desai et al. found that ανβ3, a marker of angiogenesis, exhibited elevated immunoreactivity, and vascular densities were increased in the hippocampus, midfrontal cortex, and other brain regions of AD cases compared to controls (Desai et al., 2009). The above studies on vascular densities did not use accurate quantitative methods. To our knowledge, Lee et al. used stereological methods to investigate the capillaries in the corpus callosum of AD mice for the first time, and they reported that, compared to the control mice, the capillary number in the corpus callosum of AD mice was decreased by 21%, and the total length of the capillaries in the corpus callosum of AD mice was not changed (Lee et al., 2005). However, from the methodological point of view, capillary number could not have been obtained with their method. In fact, they obtained the capillary profiles in the corpus callosum. Our results quantify the total absolute value of parameters of the capillaries other than density value, which can avoid the problems related to the "reference space trap" induced by the density values. Second, capillary distributions in the white matter were not of random orientation; this type of distribution may cause a potential estimation bias. In the current study, we used the isector technique to guarantee isotropic and uniform, random sections so capillaries had the same probability of being sampled in every direction of 3D space (Nyenggard & Gundersen, 1992). Therefore, this study provided accurate stereological data of the white matter capillaries in the early‐stage AD mice. Moreover, the current methods can be used to study postmortem human AD brain. When the methods are used in human brain, the researcher needs to adjust the sampling interval according to human brain. There were several differences between our study and the study of Lee et al. First, Lee et al. estimated the capillary number and the total capillary length in the corpus callosum. In contrast, we estimated the geometric parameters of the capillaries in the white matter, such as the total volume of the capillaries, the total surface area of the capillaries, and the total length of capillaries in the white matter. Furthermore, Lee et al. calculated the capillaries in the corpus callosums of 7‐month‐old AD mice, while we calculated the capillaries in the white matter at the age of 10 months because our previous study found that behavioral changes in AD mice began at 10 months (Zhang et al., 2013). In a clinical study, Challa et al. found that the string vessels were increased in the white matter of AD patients, which suggested a decreased of the vascular supply in the white matter of AD (Challa, Thore, Moody, Anstrom, & Brown, 2004). The significance of animal research is the ability to systematically quantify the white matter vessels of AD. Moreover, in animal research, it is possible to conduct research in the early stage of AD, which is very important for the prevention of AD. Using dynamic susceptibility contrast magnetic resonance imaging, Eskildsen et al. studied microvascular blood flow in white matter without damaging the brain (Eskildsen et al., 2017). Our stereological vascular study is a quantitative study of the internal structure of blood vessels, which is more reflective of the details of AD‐damaged blood vessels than just local blood flow. In the future, we need to figure out how the stereological methods can be used together with the image techniques so that the microvascular changes in vivo can be studied.

The marked changes in the capillary microcirculation in the white matter might induce an insufficient blood supply to the white matter in AD. The findings of a previous study indicated that the vascular changes were related to white matter lesions (Brown et al., 2000). Using the imaging techniques, researchers found that the white matter volume in AD patients was decreased (Guo et al., 2010; Medina et al., 2006; Salat et al., 2009). In contrast, our results showed that there was no significant difference in the white matter volume between the Tg2576 AD mice and the wild‐type group mice at 10 months of age. Even though we could not directly compare our results in the AD mice with the previous findings in human AD, we suspected that the following reasons might partly explain the difference. First, the results from AD patients were from aged subjects, while our results were from the early‐stage AD mice. Second, the current study used three‐dimensional quantitative methods, namely, the stereological methods, while, all of the above imaging studies (Guo et al., 2010; Medina et al., 2006; Salat et al., 2009), from a methodological point of view, were qualitative or semiquantitative. Combining the current results with our previous results, we could see that the capillary microcirculation changes were present before the white matter atrophy and the myelinated fiber changes in the white matter of Tg2576 AD mice, and we suggested that the capillary microcirculation changes might play a vital role in the white matter lesions in early‐stage AD. The results of a previous study indicated that white matter changes predicted incident AD in early stages (Brickman et al., 2012). What is more, studies have shown that the white matter pathological changes before the deposition of amyloid plaque and neurofibrillary tangles formed in gray matter in AD animal models (Redwine et al., 2003). The previous results mentioned above, together with our current results and our previous results, suggest that capillary microcirculation changes in the white matter may be one of the earliest changes in the AD brain, and they might play a vital role in the progression of AD.

## 5 CONCLUSION

In conclusion, the pathological progression of cognitive decline in AD mice was associated with the capillary changes within the white matter. The capillary microcirculation changes were present before white matter atrophy and the myelinated fiber changes in the white matter of AD mice. The current results might provide structural basis for understanding the pathological changes of the early stage of AD. Capillaries within the white matter might, thus, serve as a valid target for the prevention and treatment of early‐stage AD.

## ETHICAL APPROVAL

This study was approved by the Animal Care and Research Committee of Chongqing Medical University, China.

## CONFLICTS OF INTEREST

The authors declare that there is no conflict of interest regarding publication of this article.

## AUTHOR CONTRIBUTIONS

Conception and design of the study: Yi Zhang, Yong Tang; Acquisition and analysis of data: Yi Zhang, Chunni Zhou, Lin Jiang, Fenglei Chao; Drafting a significant portion of the manuscript or figures: Yi Zhang, Fenglei Chao; Lei Zhang, Linmu Chen, Wei Lu, Rong Jiang, Yong Tang.
